# Quantifying Total Influence between Variables with Information Theoretic and Machine Learning Techniques

**DOI:** 10.3390/e22020141

**Published:** 2020-01-24

**Authors:** Andrea Murari, Riccardo Rossi, Michele Lungaroni, Pasquale Gaudio, Michela Gelfusa

**Affiliations:** 1 Consorzio RFX (CNR, ENEA, INFN, Universita’ di Padova, Acciaierie Venete SpA), Corso Stati Uniti 4, 35127 Padova, Italy; 2 Associazione EURATOM-ENEA, University of Rome “Tor Vergata”, 00133 Rome, Italy; riccardo.rossi.en@gmail.com (R.R.); Michele.lungaroni@uniroma2.it (M.L.); gaudio@ing.uniroma2.it (P.G.); gelfusa@ing.uniroma2.it (M.G.)

**Keywords:** machine learning tools, information theory, information quality ratio, total correlations, encoders, autoencoders

## Abstract

The increasingly sophisticated investigations of complex systems require more robust estimates of the correlations between the measured quantities. The traditional Pearson correlation coefficient is easy to calculate but sensitive only to linear correlations. The total influence between quantities is, therefore, often expressed in terms of the mutual information, which also takes into account the nonlinear effects but is not normalized. To compare data from different experiments, the information quality ratio is, therefore, in many cases, of easier interpretation. On the other hand, both mutual information and information quality ratio are always positive and, therefore, cannot provide information about the sign of the influence between quantities. Moreover, they require an accurate determination of the probability distribution functions of the variables involved. As the quality and amount of data available are not always sufficient to grant an accurate estimation of the probability distribution functions, it has been investigated whether neural computational tools can help and complement the aforementioned indicators. Specific encoders and autoencoders have been developed for the task of determining the total correlation between quantities related by a functional dependence, including information about the sign of their mutual influence. Both their accuracy and computational efficiencies have been addressed in detail, with extensive numerical tests using synthetic data. A careful analysis of the robustness against noise has also been performed. The neural computational tools typically outperform the traditional indicators in practically every respect.

## 1. Quantifying the Influence between Variables

Causality is an essential element of human cognition and is typically the final goal of most scientific enterprises. Correlation is not necessarily the same as causality but assumes, nonetheless, great importance in itself or as a prerequisite to determining causal influences between quantities. Therefore, accurately quantifying the correlation between variables remains an important task in a wide range of intellectual disciplines. On the other hand, particularly when analyzing cross-sectional data, even the preliminary stage of determining the correlation between quantities can become a very challenging task. This is particularly true in the investigation of complex systems in the presence of overwhelming amounts of data. Modern scientific experiments are meant to provide information about more complex everyday phenomena. They can also produce very large amounts of data. The Joint European Torus (JET) has the potential of producing 1 terabyte of data per day, while A Toroidal LHC ApparatuS (ATLAS) can generate more than 10 petabytes of data per year [1]. 

Given this data deluge, the need to accurately assess the relation between the various measured quantities has become more pressing. The probability that important information remains hidden in the large data warehouses is indeed very high. The other main risk resides in the possible wrong evaluation of the influence between variables, which could lead to significant blunders [2]. 

The most widely used tools to determine the relation between variables present some significant limitations; they either detect only the linear correlations or require large amounts of data and do not provide any hint about the directionality of the influence (see Section 2). In this contribution, it is explored to what extent specific neural networks can help in at least alleviating some of these insufficiencies. The developed tools, introduced in Section 3, aim to determine the influence between quantities linked by a functional relation, typically non-linear, in the case of multivariate problems and in presence of significant noise. A comprehensive series of numerical tests with synthetic data has been performed. The results are reported in Section 4 for the linear correlations. The potential of the developed techniques to quantify total correlations is discussed in Section 5 for the case of a single regressor, including a detailed analysis of the effects of the noise. The application of the tools to multivariate problems is investigated in Section 6. Conclusions and lines of future investigation are the subject of the last section of the paper. 

## 2. Correlation and Mutual Information between Variables

A very common and computationally efficient indicator to quantify the linear correlations between variables is the Pearson correlation coefficient (PCC). The PCC has been conceived to determine the bivariate correlations between two quantities in the available data sets. The definition of the PCC, traditionally indicated by the Greek letter *ρ*, is

(1) $$\rho_{X,Y}=\frac{\mathrm{cov}\left(X,Y\right)}{\sigma_{X}\sigma_{Y}},$$

where cov is the covariance and σ indicates the standard deviation of the variables. In the notation adopted in this paper, the variable *Y* is the dependent variable and *X* are the regressors.

The main drawback of the PCC resides in the fact that it takes into account only the linear correlations. In the case of investigations involving highly nonlinear phenomena, as it is often the case in the science of complex systems, the conclusions obtained from the analysis of the PCC can, therefore, be highly misleading. To overcome this limitation, unbiased techniques for non-linear analysis are required. Information theory provides a very powerful tool to investigate the information transfer between quantities, the so-called mutual information [3]. This indicator can be considered a measure of the mutual dependence between two random variables *X* and *Y*; it quantifies the amount of information that can be obtained about one random variable from knowing a second random variable and includes nonlinear effects. The traditional symbol for the mutual information is *I*(*X*,*Y*), which, for discrete variables, is defined as

(2) $$I\left(X,Y\right)=-\sum_{x}\sum_{y}P\left(x,y\right)\ln\left(\frac{P\left(x,y\right)}{P\left(x\right)P\left(y\right)}\right),$$

where *P*() indicates the probability density function (pdf) of the variables in the brackets. Unfortunately, the mutual information is not a normalized quantity. On the other hand, it can be rescaled to the interval [0–1], by dividing it by the joint entropy *H(X,Y)* defined as

(3) $$H\left(X,Y\right)=-\sum_{x}\sum_{y}P\left(x,y\right)\ln P\left(x,y\right).$$

In the literature, this new normalized quantity is called the information quality ratio (IQR) [3]:

(4) $$IQR=\frac{I\left(X,Y\right)}{H\left(X,Y\right)}.$$

The information quality ratio is a well-consolidated quantity, but, as can be seen from Equations (2) and (3), the estimation of the IQR requires the calculation of the probability density functions of the variables involved. This aspect renders the analysis more demanding, compared to the PCC, both in terms of efforts and requirements about the quality of the data. Fortunately, nowadays, quite powerful tools exist for obtaining the pdfs from the histograms of experimental data. The techniques used in this paper belong to the family of the “kernel density estimation” (KDE) [4], which have also been deployed in [5,6,7,8,9] for the analysis of experimental data from Big Physics experiments, using the methodology described in [10,11]. The outputs of these KDE tools can be considered consolidated at this stage. However, density estimation remains a delicate operation. Moreover, the IQR is always a positive quantity and, therefore, does not shed any light about the directionality of the information transfer and, therefore, of the influence between the considered quantities. 

Given the previous considerations, it is reasonable to ask whether neural computation can help to overcome or, at least, alleviate the aforementioned limitations of PCC and IQR. In this perspective, in the framework of deep learning for image processing, many neural network topologies, such as encoders and autoencoders, have been recently extensively tested (see next section for a description of these networks); their properties could, in principle, also be useful in the analysis of the correlation between quantities [12]. In particular, it is worth assessing to what extent encoders and autoencoders can provide information about the total correlations between quantities, including directionality, and whether they can be more computationally efficient than information theoretical indicators based on density estimators.

## 3. The Technology of Autoencoders and Encoders for the Assessment of Correlations

**Figure 1:**
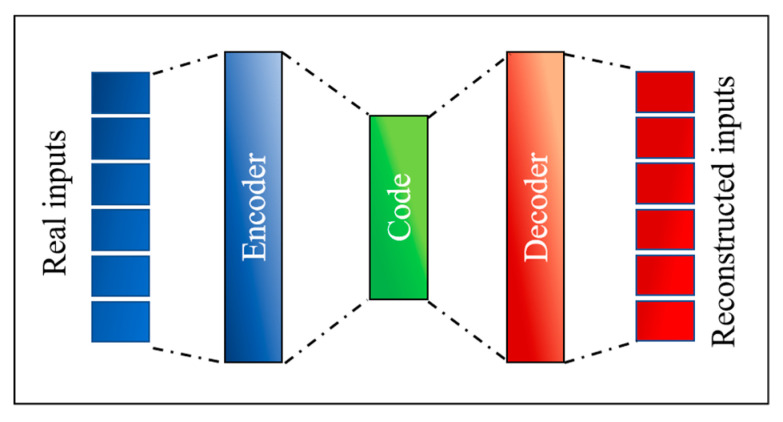
General topology of autoencoders.

Autoencoders are feedforward neural networks with a specific type of topology, reported in Figure 1. The defining characteristic of autoencoders is the fact that the output is the same as the input [12]. They are meant to compress the input into a lower-dimensional code and then reconstruct the output from this representation. To this end, an autoencoder consists of three components: Encoder, code, and decoder. The encoder compresses the input and produces the code, a more compact representation of the inputs. The decoder then reconstructs the input using this code only. The code constitutes a compact “summary” or “compression” of the input, which is also called the latent-space representation.

In more detail, the input passes first through the encoder, which is a fully connected artificial neural network (ANN), and is translated into a code. The code is the input of the decoder, which is meant to produce an output, which is as similar as possible to the input. The decoder architecture is typically the mirror image of the encoder. Even if this condition is not an absolute requirement, it is typically the case (the actual indispensable requirement is that the dimensionality of the input and output is the same). Autoencoders can be trained via backpropagation as traditional ANNs.

**Figure 2:**
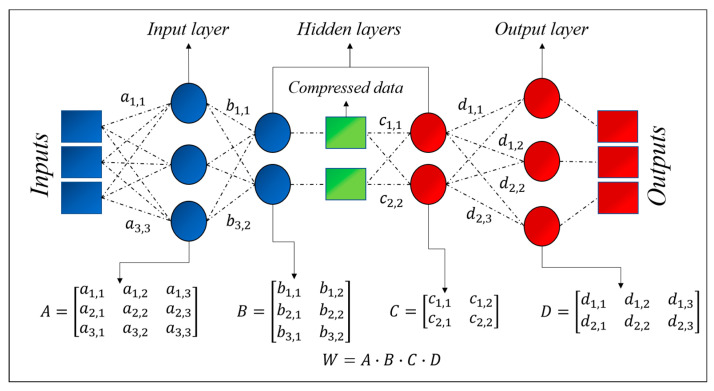
Architecture of the autoencoders used in the present work with the matrixes that are multiplied to give the matrix W. The case shown in the figure is particularized for a latent space of dimension 2.

Overall, there are four hyperparameters that need to be to set before starting the training of an autoencoder: (1) Code size, i.e., the number of nodes in the middle layer; (2) the number of layers; (3) the number of nodes per layer; (4) the loss function. All these hyperparameters have to be set to optimize the autoencoders for the present application, i.e., the assessment of the total correlation between variables (inputs). To this end, a stacked autoencoder architecture has been adopted: The layers are stacked one after another. The actual architecture of the autoencoders implemented to obtain the results discussed in the next sections is reported in Figure 2. For the investigations reported in this paper, linear activation functions have been implemented.

The architecture of the autoencoders is well suited to the determination of the total correlation between quantities. Another important type of correlation is the one between regressors and dependent variables. This is the case of regression, a very relevant application for both scientific and engineering studies. For this type of study, the topology of the so-called encoders is sufficient. Encoders can be thought of as just the first part of the autoencoder (see Figure 1) with the code of the same dimensionality as the dependent variable space. 

The basic elements of the proposed method, to obtain the correlations (linear or total), consists of adopting the architecture of Figure 2 (or of the simple encoder for the case of regression) and then of reducing the neurons in the intermediate layer until the autoencoder does not manage to reproduce the outputs properly (starting with a number of neurons equal to the number of inputs). After identifying the dimensionality of the latent space, the minimum number of neurons in the intermediate layer, for which the inputs are properly reconstructed at the output, a specific manipulation of the weights allows the required information to be obtained. The operations of the weights differ depending on whether the linear or nonlinear correlations are investigated, and, therefore, the details are provided in the next sections.

## 4. The Technology of Autoencoders and Encoders for the Assessment of Linear Correlations

Even if the main objective of the work consists of finding an alternative method to quantify the total correlations between quantities, a first analysis of the linear correlations is worth the effort. Being able to reproduce the PCC is useful to grasp the main elements of the approach and also to increase the confidence in the results. On the other hand, the PCC can be strongly affected by noise present in the data; assessing whether neural computational tools can help in this respect is, therefore, quite valuable, particularly for scientific applications such as thermonuclear plasmas, whose measurements present quite high levels of uncertainties. 

To fix the ideas, let us consider two examples involving three variables *x*_1_, *x*_2_, and *x*_3_. In the first case, the correlation between the first two variables is of value unity (*x*_2_ = *cost* *x*_1_); in the second case, the correlation is reduced to 0.8 by the presence of random noise (*x*_2_ = *cost x*_1_ + *random*), whose standard deviation is 20% of the actual signal standard deviation. In both examples, the dimensionality of the latent space is 2. The matrix of the weights of the autoencoders can be expressed in matrix form as

(5) $$W=\left[\begin{array}{ccc} W_{1,1} & W_{1,2} & W_{1,3}\\ W_{2,1} & W_{2,2} & W_{2,3}\\ W_{3,1} & W_{3,2} & W_{3,3} \end{array}\right].$$

The derivation of the matrix Λ for this specific case of dimensionality 2 is reported in Figure 2. Extension to latent spaces of higher dimensionality is straightforward. To obtain normalized coefficients, so that the correlation coefficient of a variable with itself is 1, it is necessary to define a new matrix Λ, whose coefficients are

(6) $$\Lambda_{i,j}=\sqrt{\frac{2W_{i,j}W_{j,i}}{W_{i,i}^{2}+W_{j,j}^{2}}}.$$

**Figure 3:**
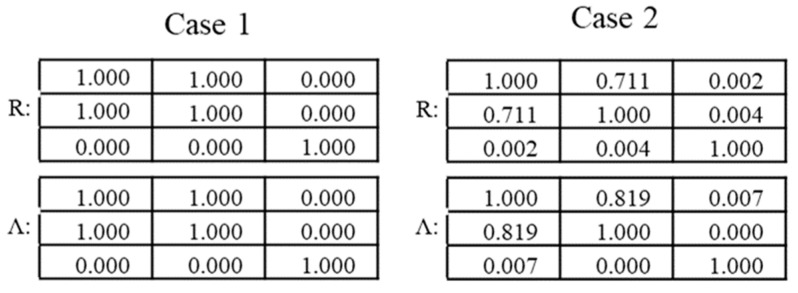
Comparison of correlation coefficients for the two examples described in the text. Case 1: No noise. Case 2: Gaussian noise with standard deviation of 20% of the actual signal standard deviation.

The tables reported in Figure 3 provide a comparison of the correlation coefficients calculated with the PCC and with the proposed method of the autoencoders. As can be concluded by simple inspection of the numerical values in these tables, the proposed technique manages to exactly reproduce the PCC estimates in the case of the absence of noise. When applied to noisy entries, the autoencoder provides a better estimate of the expected off-diagonal correlation coefficients, in the sense that they are closer to 1, the original correlation between the variables before adding the noise. This is an interesting point, which will be analyzed more extensively at the end of this section.

A series of numerical tests has been performed to prove the generality of the conclusions obtained for simple cases. For example, it has been verified that the approach remains valid for problems of larger dimensionality. An example of 10 variables is reported in the following. A set of 10 different variables have been generated: *x*_1_, *x*_2_, *x*_3_, *x*_4_, *x*_5_, *x*_6_, *x*_7_ are independent from each other. The remaining variables have been generated with the relations: *x*_8_ = *cost*_1_ *x*_1_; *x*_9_ = *cost*_2_ *x*_2_; *x*_10_ = *cost*_3_ *x*_3_.

**Figure 4:**
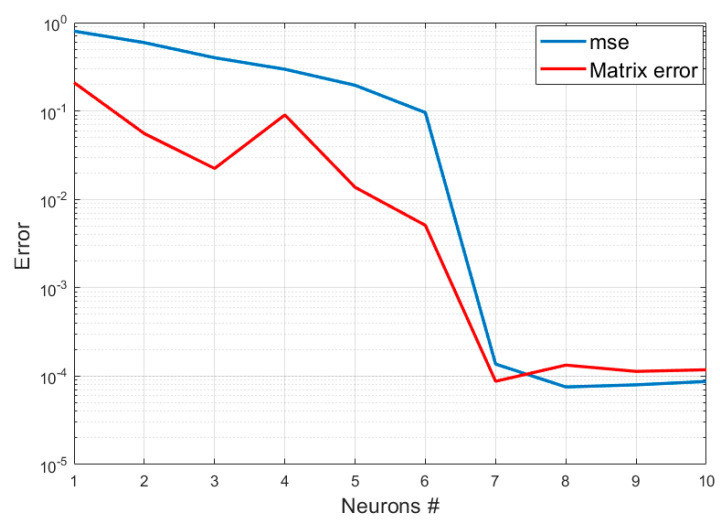
Trend of the errors in the reconstruction of the input data with the dimensionality of the intermediate layer in the autoencoder.

**Figure 5:**
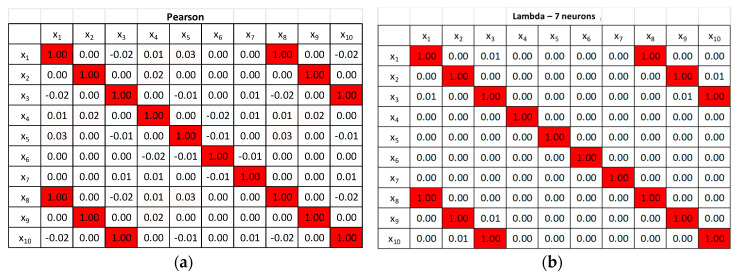
(**a**) The Pearson correlation coefficient (PCC) for a set of 10 variables correlated as specified in the text. (**b**) The correlation coefficients obtained with the proposed method of the autoencoders.

As can be seen in the plots of Figure 4, the autoencoder manages to clearly identify the dimensions of the latent space. The minimum number of neurons in the intermediate layer, for which the autoencoder manages to reproduce the inputs with minimal errors, is 7. The matrix Λ of the correlation coefficients also reproduces exactly that obtained with the application of the PCC, as shown in Figure 5.

**Figure 6:**
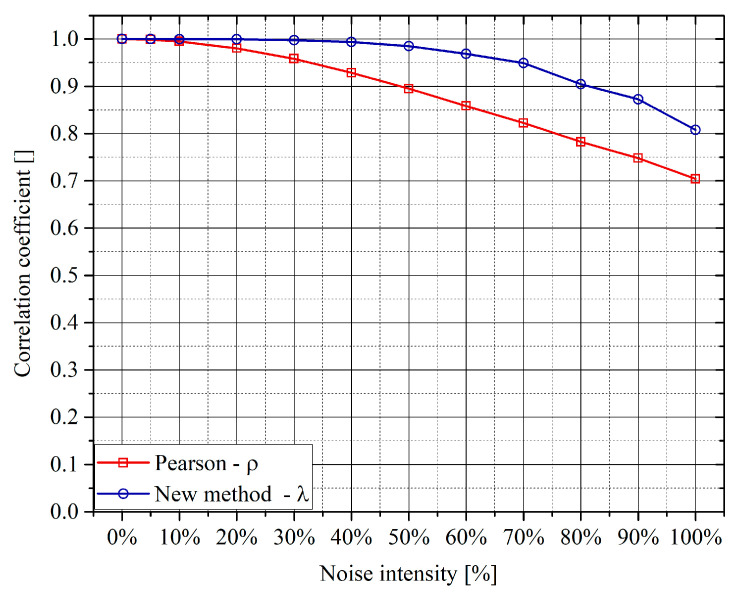
Trend of the off-diagonal term of the matrix Λ and the PCC versus the percentage of additive Gaussian noise. The noise intensity is calculated as the standard deviation of the noise divided by the standard deviation of the variable amplitude.

Systematic tests have also been performed to assess the robustness of the proposed approach to additive noise. Gaussian noise of various amplitudes has been added to the variables. It is important to notice that the method based on the autoencoders has proved more resilient that the traditional PCC. In general, for linear correlations, the Pearson coefficient starts declining for a standard deviation of the noise of the order of 20% of the signal amplitude, whereas the matrix Λ remains stable up to even 60% of additive noise. A typical dependence of the off-diagonal terms of the matrix Λ and the traditional PCC, versus the percentage of noise, is shown in the plots of Figure 6. Of course, the better robustness of the networks becomes important when multivariate linear correlations have to be analyzed, as, in those cases, it is not obvious how to separate the influence of other quantities from the effects of the noise. 

In the case of regression, when the objective consists of finding the correlation between one dependent variable and a series of regressors, the analysis must be performed in two uncorrelated steps. The linear correlations between the regressors can be determined as just described previously in this section. Then, the linear correlation between the dependent variable and each of the regressors has to be determined directly with a specific network, completely independent from the previous one. The reason why the analysis cannot be performed in a single step, using one single network, is that the dependent variables create spurious influences between the regressors, which are not the desired output of the analysis. As a simple case, just to exemplify the problem, let us assume that both *x*_1_ and *x*_2_ influence *y*. The neural tools have no way to distinguish between the dependent variable and the regressors. Therefore, the encoder may also find a correlation between *x*_1_ and *x*_2_, through y, even if *x*_1_ and *x*_2_ are completely independent. The two-step approach is the only simple and effective solution found so far. The output for a representative case with the functional dependence *y* = –3*x*_1_ + 4*x*_2_ is reported in Table 1.

## 5. Numerical Tests for Total Correlations: The Bivariate Case

The quantification of the total correlation between measurements poses some additional problems. First, if the influence between the quantities under investigation includes nonlinear effects, the level of correlation depends on the range of the variables themselves. A simple example is a parabolic dependence, which is not only constant but whose sign also depends on the range of the variables. To address this issue, the procedure devised for assessing the linear correlations with the autoencoders has to be modified as follows. The determination of the latent space is the same. Once this step is completed, it is necessary to subdivide the range of the variables in sufficiently small intervals; on every one of these intervals, the local correlation coefficient between the quantities of interest can be calculated as described in the previous section. The integral of these local correlation coefficients is the integral correlation indicated with *ρ_int_*:

(7) $$\rho_{int}=\frac{1}{\Delta x}\int \left|\rho \left(x\right)\right|dx.$$

Of course, as the IQR, this indicator does not provide any information about the sign of the correlation. To quantify this aspect, a good indicator is the monotonicity of the correlation, which can be defined as

(8) $$M_{int}=\frac{1}{\Delta x}\int sign\left(\rho \left(x\right)\right)dx.$$

**Figure 7:**
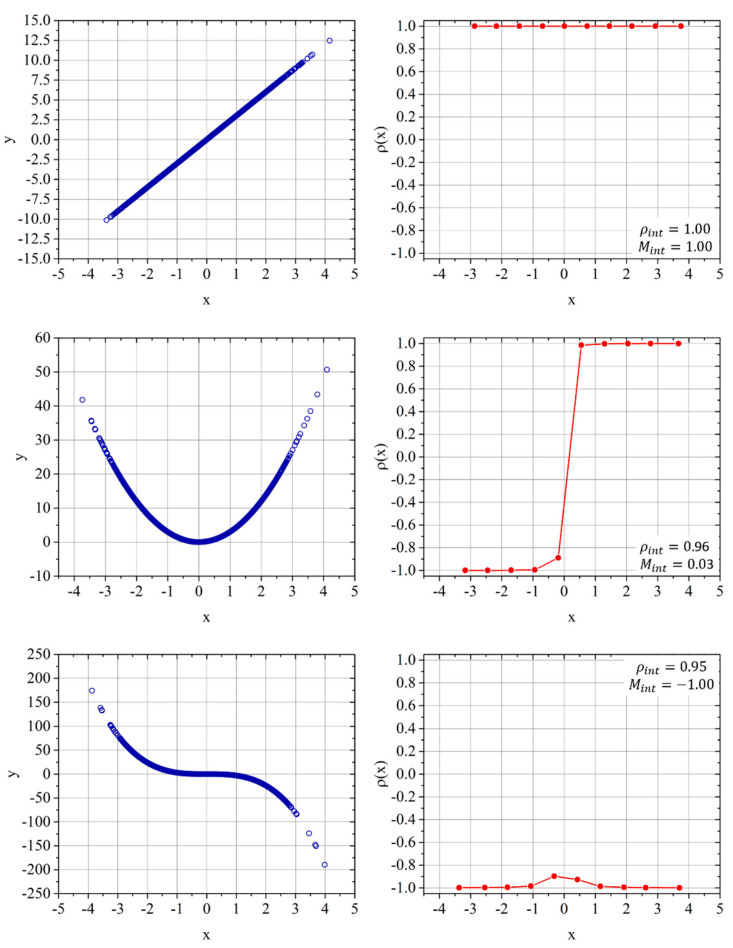
**Top**: Two linearly dependent variables (**left**) and the relative local correlation coefficient *ρ_int_* (**right**). **Middle**: Two quadratic-dependent variables (**left**) and the relative local correlation coefficient *ρ_int_* (**right**). **Bottom**: Two variables with a negative cubic dependence (**left**) and the relative local correlation coefficient *ρ* (**right**). The integral values of the correlation coefficient and of the monotonicity are reported in the insets.

To exemplify the potential of the proposed approach, Figure 7 reports the local correlation coefficient for a linear, quadratic, and cubic dependence. For the first case, as expected for a linear dependence, both the integral correlation coefficient and the monotonicity have a value of one. For the quadratic case, the *ρ_int_* is again practically unitary, whereas the monotonicity is almost zero. In the cubic case, the *ρ_int_* is again unity and the monotonicity −1. Of course, for these nonlinear dependencies, the PCC fairs quite poorly; it is practically zero for the quadratic case and about −0.9 for the cubic function.

The combination of the integrated correlation coefficient and the monotonicity is, therefore, much more informative than the simple IQR. The *ρ_int_* represents very well the actual dependence between the variables. Moreover, the monotonicity provides information about the direction and the constancy of the mutual influence; negative signs of the monotonicity indicate an inverse dependence, and low values denote the fact that the mutual dependence changes sign over the domain of the variables. 

**Figure 8:**
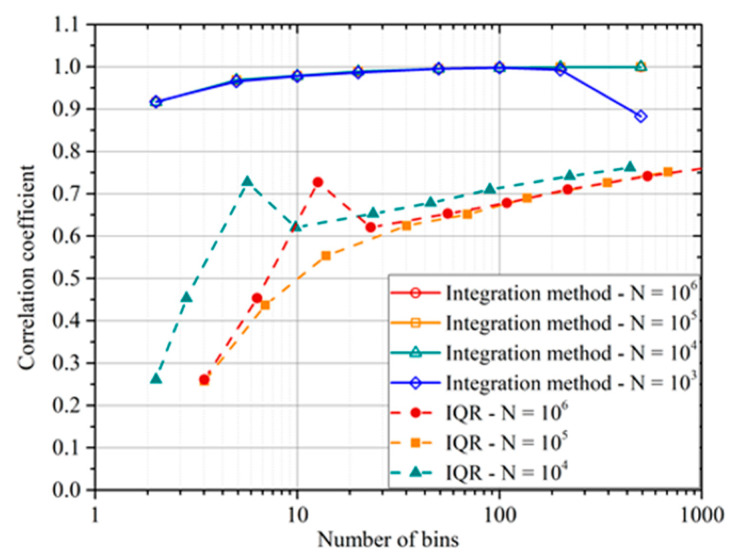
Comparison of the *ρ_int_* and the IQR for the negative cubic dependence (third case of Figure 7). The *x*-axis reports the number of bins and N is the number of generated points used to calculate the indicators.

In terms of comparison with the IQR, Figure 8 summarizes a typical trend with the number of bins and the number of the entries in the database. As can be concluded from simple inspection of the plot, *ρ_int_* provides a much better estimate of the correlation level between the independent and dependent variables (the actual value in the synthetic data is 1). The integrated correlation coefficient is also much more robust against the choice of the bins and the number of entries, two factors that affect a lot the IQR that is based on the details of the pdf. 

**Figure 9:**
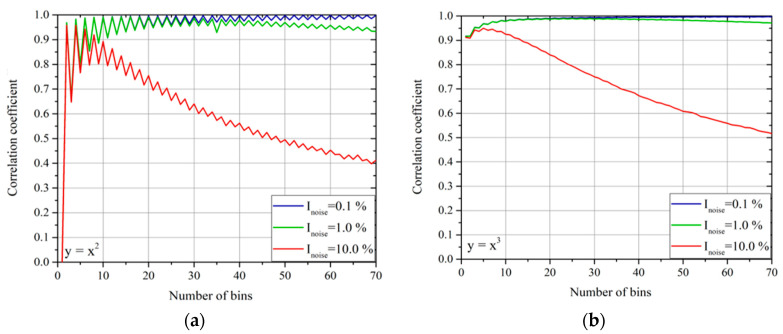
Effects of the noise amplitude on *ρ_int_* for various choices of the number of bins. (**a**) The investigated dependence is *y* = *x*^2^. (**b**) the investigated dependence is *y* = *x*^3^. The independent variable *x* varies in the range [–10;10] and the number of points is 10^5^. The noise intensity is calculated as the standard deviation of the noise divided by the standard deviation of the variable amplitude.

In addition, for the total correlation of bivariate dependencies, a careful analysis of the noise effects has been carried out. In this case, the situation is a bit more involved than for the linear correlations. In particular, the effect of the noise on *ρ_int_* does not depend only on its amplitude but also on the choice of the bins for the determination of the pdfs and the integral correlation. Representative examples, showing the trends with noise amplitude and choice of the bins, are reported in Figure 9. The plots of this figure refer to the Gaussian noise of amplitude, proportional to the signal amplitude; as usual, the noise intensity is expressed as the standard deviation of the noise divided by the standard deviation of the variable amplitude.

As can be derived from inspection of Figure 9, the choice of the bins is crucial. It is indeed important that a sufficient number of points are included in each bin, so that the detrimental effects of the noise can be averaged out by the statistics. Thus, a sufficiently low number of bins is to be preferred to ensure compatibly with the need to properly resolve the variations in ρ_int_. If the number of data is not sufficient but the properties of the noise are known, it has been verified that bootstrapping can be usefully implemented to obtain reasonable values of *ρ_int_*. The oscillations in the value of *ρ_int_* for the quadratic dependence are a consequence of the switch between an even and odd number of bins. In the case of an even number of bins, the central one is centered around zero and, therefore, gives a null correlation, reducing the total value of *ρ_int_* compared to the case of odd bins. Again, this is a problem, which can be easily alleviated by bootstrapping. It is also worth mentioning that, contrary to *ρ_int_*, the monotonicity is extremely robust against both the choice of the bins and the level of the noise (even in the range of 10–20% of the signal amplitude). Moreover, it has been checked that M_int_ gives practically the same results as Kendall’s correlation coefficient, an indicator traditionally used for the same purpose [13,14]; the monotonicity criterion proposed in this paper is even less sensitive to noise, a quite interesting property given the well-known robustness of Kendall’s correlation coefficient. 

## 6. Numerical Tests for Total Correlations: The Multivariate Case

Conceptually, the extension of the methodology, presented in the previous section, to the investigation of multivariate problems does not pose any principal difficulty. Equations (7) and (8) can be generalized easily to obtain

(9) $$\rho_{int}=\frac{1}{\Delta x_{1}\Delta x_{2}\ldots \Delta x_{N}}\int \left|\rho \left(x_{1},x_{2},\ldots ,x_{N}\right)\right|dx_{1}dx_{2}\ldots dx_{N},$$

(10) $$M_{int}=\frac{1}{\Delta x_{1}\Delta x_{2}\ldots \Delta x_{N}}\int sign\left(\rho \left(x_{1},x_{2},\ldots ,x_{N}\right)\right)dx_{1}dx_{2}\ldots dx_{N}.$$

Again, the only precaution is that, in the case of regression, the analysis must be performed in two steps as for the investigation of the linear multivariate correlations. The reason is also the same, i.e., the need to avoid spurious influences between the regressors introduced by the dependent variable. To show the potential of the method, the results of a representative example are reported in Table 2 and Table 3. The functional dependence used to generate the synthetic data is $y=3x_{1}^{2}-x_{2}^{3}$. The range of xi is [−10;10] and the number of points is 105.

With regard to the competitive advantages of the neural computational tools, they remain the same as those discussed for the univariate cases. 

## 7. Conclusions

An approach to the identification of the mutual influence between variables, using neural computational tools, has been proposed. The developed techniques have been validated with a series of systematic tests with synthetic data. The use of autoencoders and encoders has provided very interesting results. For the determination of the linear correlations between quantities, the proposed method provides the same values as the PCC but is significantly more robust against the effects of additive random noise. To investigate the total correlations between quantities, the combined use of the integrated correlation coefficient and the monotonicity has proved to be much more informative than the IQR. The *ρ_int_* reflects quite well the actual dependence between quantities. The monotonicity provides very valuable information about the constancy of the mutual influence over the investigated domain. The *ρ_int_* is also less sensitive to the details of parameters, mainly the number of bins, required to calculate IQR. The *ρ_int_* is also less demanding in terms of quantity and quality of the data required, to provide reliable estimates of the mutual influence between quantities. Indeed, all the numerical tests performed reveal that *ρ_int_* is more robust against noise and, other things being equal, that it requires fewer entries to converge on accurate results.

With regard to future developments, the main issue to investigate is the requirement in terms of data quality and volume for the case of multivariate nonlinear dependencies, once the number of regressors is very high (of the order of 10 or more). On a longer time perspective, it is intended to explore the potential of the proposed neural networks to address more complex situations, such as the existence of data clusters, general (non-functional) constraints in specific regions of the domain, and more complex noise models than unimodal/Gaussian.

## Figures and Tables

**Table 1:** Comparison of the linear correlations for the equation *y* = –3*x*_1_ + 4*x*_2_ obtained with the PCC and the neural computation tools. The variable *x*_3_ has been randomly generated and has no correlation (expected correlation with the third variable is zero) with the other two. Red color mean a positive correlation, while blue color means negative. Color intensities are proportional to correlation and white is a null correlation.

	*x*_1_	*x*_2_	*x*_3_	*y*
***Pearson***	−0.60	0.80	0.00	1.00
***Ρ_networks_***	−0.59	0.80	0.08	1.00

**Table 2:** Total correlations between the regressors for the functional dependence $\;y=3x_{1}^{2}-x_{2}^{3}$. Red color mean a positive correlation, while blue color means negative. Color intensities are proportional to correlation and white is a null correlation.

	*x*_1_	*x*_2_	*x*_3_
***x_1_***	1.00	0.05	0.03
***x_2_***	0.02	1.00	0.01
***x*_3_**	0.02	0.03	1.00

**Table 3:** Total correlations between the output and the regressors for the functional dependence $\;y=3x_{1}^{2}-x_{2}^{3}$. Red color mean a positive correlation, while blue color means negative. Color intensities are proportional to correlation and white is a null correlation.

	*x*_1_	*x*_2_	*x*_3_	*y*
***ρ_int_***	0.46	0.75	0.08	1.00
***M***	0.02	−0.96	0.01	1.00
